# Human Metapneumovirus-associated Hospital Burden in Older Adults in Scotland: A Retrospective Analysis

**DOI:** 10.1093/ofid/ofag057

**Published:** 2026-02-11

**Authors:** Durga Kulkarni, Richard Osei-Yeboah, Kate Templeton, Harish Nair

**Affiliations:** Centre for Global Health, Usher Institute, University of Edinburgh, Edinburgh, UK; Centre for Global Health, Usher Institute, University of Edinburgh, Edinburgh, UK; Viral Genotyping Reference Laboratory Edinburgh, NHS Lothian, Royal Infirmary of Edinburgh, Edinburgh, UK; Centre for Global Health, Usher Institute, University of Edinburgh, Edinburgh, UK; School of Public Health, Nanjing Medical University, Nanjing, China; MRC/Wits Rural Public Health and Health Transitions Research Unit (Agincourt), School of Public Health, Faculty of Health Sciences, University of the Witwatersrand, Johannesburg, South Africa

**Keywords:** hMPV, hospital admissions, respiratory tract infections, United Kingdom

## Abstract

**Background:**

Human metapneumovirus (hMPV) is commonly associated with respiratory tract infections (RTIs) in older adults.

**Method:**

We estimated the annual hospital incidence of hMPV RTIs in older adults in Scotland over 6 seasons (2017–2023) using national hospital and laboratory data. Hospital incidence in Scottish health boards other than Lothian, where testing practices were uncertain, was extrapolated using Lothian's comprehensive laboratory data. We also assessed the severity and mortality outcomes of laboratory-confirmed hMPV episodes in older adults in Scotland. Additionally, we developed similar estimates for respiratory syncytial virus (RSV) and Influenza A for comparisons.

**Results:**

This analysis included 626 laboratory-confirmed hMPV hospitalizations. Only 28% of laboratory-confirmed hMPV episodes were clinically coded. The extrapolated hMPV hospital incidence ranged from 3.6/100 000 to 49.5/100 000 in adults aged ≥60 y in Scotland, and the extrapolated incidence was 1.3 to 3.8 times higher than the incidence of laboratory-confirmed data. The hospital incidence was higher in ≥75 y than in 60–74 y. hMPV incidence dropped substantially during the COVID-19 pandemic. The case fatality rates were higher in the ≥75 y compared with the 60–74 y, but ICU admissions were more common in the 60–74 y. The hospital incidence of RSV and Influenza A seemed higher than hMPV, but the 95% confidence intervals for severity and outcomes generally overlapped.

**Conclusions:**

hMPV RTIs pose a substantial hospital burden in older adults in Scotland. The differences between laboratory-confirmed and extrapolated estimates highlight the need for improved surveillance, diagnosis, and coding practices to develop robust burden estimates. The clinical severity of hMPV could be similar to RSV.

Human metapneumovirus (hMPV) is a common respiratory virus associated with respiratory tract infections (RTIs) in humans. Vaccine candidates targeting hMPV, in combination with other respiratory viruses like respiratory syncytial virus (RSV) or Parainfluenza virus, are currently undergoing testing in clinical trials [1–3]. Consequently, a good understanding of hMPV epidemiology is essential to inform future immunization policies. On a separate note, an increase in hMPV cases from China in early 2025 had raised concerns about an emerging pandemic, but no alteration in the pathology of hMPV infections was noted [4]. Nonetheless, ongoing monitoring and surveillance are critical for detecting atypical epidemiological patterns.

Young children aged ≤2 years (≤2 y), older adults aged ≥60 y, and immunocompromised persons are at high risk for hMPV RTIs [5, 6]. We previously estimated about 473 (95% CI: 396–777) thousand hMPV-associated hospitalizations globally and 185 (105–340) thousand in high-income countries in persons aged ≥65 years in 2019 [7].

The UK Health Security Agency recently identified hMPV as a pathogen with medium overall pandemic potential, a high overall epidemic potential, and a high domestic burden [8]. However, to the best of our knowledge, the burden of hMPV-associated RTI hospitalizations among older adults in Scotland has not been investigated.

Therefore, we conducted this study to estimate the hMPV RTI hospital burden in older adults in Scotland between July 1, 2017 and June 30, 2023. Additionally, we investigated the changes in hMPV epidemiology during the COVID-19 pandemic. We also reported comparable estimates for RSV and Influenza A for contextual reference. The results of this study will help understand resource demands, track disease patterns, and inform hMPV immunization policies in the United Kingdom in the future.

## METHODS

### Study Design and Setting

We conducted a retrospective analysis of hMPV RTI hospitalizations between July 1, 2017 and June 30, 2023, in older adults in Scotland, utilizing data from Scottish national hospital registries.

The population of Scotland was ∼5.4 million (≥60 y population ≈ 1.5 million) in 2023 [9]. Lothian represents 1 of the 14 National Health Service (NHS) Health Boards in Scotland, serving the second-largest residential population in Scotland, ∼0.9 million (≥60 y population ≈ 0.2 million) in 2023, from the councils of the City of Edinburgh, East Lothian, Midlothian, and West Lothian [9, 10]. The population in Lothian is relatively younger and ethnically more diverse than in other health boards (Supplementary Table 1) [11]. Lothian has a smaller share of its population in the 10% most deprived areas compared with the national average, suggesting that it experiences relatively lower levels of deprivation overall [12].

Scotland has a universal, publicly funded healthcare system, and healthcare, including hospital care, is free at the point of use for all Scottish residents. Influenza vaccine was recommended and offered to persons ≥65 y during the study period, with a coverage of 85.5% in this group during the 2022/23 season [13]. There were no vaccines against hMPV or RSV during the study period.

### Case Definitions

We defined older adults as persons aged ≥60 y.

RTI hospitalizations included any hospital episode with at least 1 RTI-related diagnostic code (either primary or secondary) based on the International Classification of Diseases-10th edition (ICD-10) diagnosis codes [14]. A list of eligible ICD-10 diagnostic codes is provided in Supplementary Table 2. A maximum of 6 diagnosis codes were available for each hospital episode. We included all inpatient admissions, including those lasting for <1 day, but excluded day case admissions. A day case referred to an admission to a specialty for clinical care and involved seeing a clinician (as the consultant's representative) and requiring supervised recovery in the place of treatment. Such patients are not expected to, and do not, remain overnight. Routine admissions were also excluded, as these involve planned hospitalizations unlikely to be linked to the RTI episode.

An hMPV-specific RTI hospital episode was defined as an RTI hospital episode with a positive hMPV test on a specimen collected within 7 days before and up to 3 days after the date of admission. The inclusion of a 7-day window before admission was based on the known incubation period (3 to 6 days), thus making the test results before admission relevant to the hospital admissions [15]. The 3-day window after admission was selected to allow for the inclusion of all relevant hospitalization episodes, even when the testing did not occur at the point of admission but subsequently during the hospitalization, while ruling out nosocomial infections.

The health board indicated the NHS health board in which the hospital admission occurred.

An (annual) season was defined as the period between 1 July of 1 year and 30 June of the following year, to capture all hospitalizations during the winter period.

Hospital length of stay (LOS) was defined as the difference in the number of days from the date of admission to the date of discharge. Long LOS was defined as hospital LOS >5 days [16].

Intensive care unit (ICU) admissions included ICU or high-dependency unit (HDU) admissions within the hMPV RTI hospital admission episodes. ICU admission rate referred to the proportion of hMPV RTI admissions requiring ICU (or HDU) admission and was expressed as a percentage.

The in-hospital case fatality rate (CFR) was defined as the proportion of in-hospital deaths among all hMPV RTI admissions. Similarly, the postdischarge CFR within 90 days was defined as the proportion of deaths occurring after discharge and up to 90 days postdischarge among all hMPV RTI admissions. The CFRs were also expressed as percentages.

### Data Sources and Data Linking

Hospitalization episode data were extracted from the Scottish Morbidity Records 01 (SMR01). SMR01 is an episode-based patient record related to all in-patients and day case episodes from nonobstetric and nonpsychiatric specialties in Scotland [17].

hMPV laboratory data were obtained from the Electronic Communication of Surveillance Scotland (ECOSS), a Public Health Scotland (PHS) system that collects laboratory test results, including patient identifiable data, from all NHS laboratories in Scotland [18]. We could access only positive results without data on total or negative tests. These tests were part of routine standard of care. All RTI hospital episodes in Lothian during the study period were recommended respiratory virus testing using the LUMINEX respiratory virus panel, which included hMPV, RSV, and Influenza A, as part of standard diagnostic protocol. The testing policies were variable and unclear across other health boards.

ICU or HDU admission data were sourced from the Scottish Intensive Care Society Audit Group (SICSAG), and mortality data were sourced from the National Records of Scotland (NRS) [19, 20].

All data sets were matched using pseudonymized patient identifiers that had been preassigned prior to data receipt by indexing patient data to unique identifying numbers. Furthermore, the hospital admission (SMR01), laboratory (ECOSS), and ICU (SICSAG) data were also linked according to the hospital admission date, specimen collection date (as mentioned above), and ICU admission dates (ICU admission within the hospital stay). The precise data cleaning and data linking procedures are illustrated in Supplementary Figure 1.

Population estimates required for estimating population-based rates were obtained from the NRS [9, 21]. For each season, the mid-year population estimates at the end of the season were used.

## ETHICAL APPROVAL

The Public Benefit and Privacy Panel for Health and Social Care (PBPP-HSC) approved data access to the required data in the National Safe Haven (Approval number: 2223-0019).

### Patient Consent

This research only examined existing data that were pseudonymized before analysis, and it did not require the active consent of patients. The data were collected routinely through NHS Scotland, with patients informed of potential use and their rights through various PHS and NHS Scotland privacy notices.

### Statistical Analysis

We estimated the laboratory-confirmed annual hMPV-associated RTI hospital incidence in Scotland by age bands (60–74 y and ≥75 y) for the 6 seasons. The 95% CI was estimated using nonparametric bootstrap resampling.

Given the testing policies as described above, we extrapolated for under-testing of RTI admissions in other health boards using Lothian as a benchmark. We calculated the hMPV proportion positive among all RTI-associated hospital admissions in Lothian, stratified by age bands (60–74 y and ≥75 y) and month of admission during each annual season. We applied these to the corresponding RTI admissions in other health boards. The corresponding estimates for each age band and month, in each annual season, for Lothian and other health boards were summed to generate hospital incidence estimates for the annual season. The 95% CI was estimated using nonparametric bootstrap resampling. The detailed steps are provided in Supplementary Text 1 and Supplementary Figure 2.

We conducted a sensitivity analysis and reported the laboratory-confirmed hMPV incidence in Lothian by limiting the analysis to episodes where a specimen was collected for testing within 3 days before to 3 days after the date of admission to exclude cases attributable to sequelae or complications.

We estimated the percentage of laboratory-confirmed hMPV RTI hospitalization episodes along with 95% CI that were associated with long LOS according to age bands (60–74 y and ≥75 y) in Lothian, other health boards, and Scotland. Similarly, the ICU admission rate, in-hospital CFR, and postdischarge CFR of laboratory-confirmed hMPV RTI hospitalization episodes were expressed as percentages with 95% CI according to age bands in Lothian, other health boards, and Scotland. The 95% CI were estimated using the bootstrap resampling method.

We developed similar estimates for RSV and Influenza A to provide initial insights into trends across groups.

## RESULTS

This analysis included 626 unique hospitalization episodes of laboratory-confirmed hMPV RTIs in older adults in Scotland between July 1, 2017 and June 30, 2023. The demographic characteristics of the 626 laboratory-confirmed hMPV RTIs, along with the 231 508 all-cause RTI hospitalization episodes, are summarized in Table 1. The characteristics are presented stratified by Lothian and other health boards in Supplementary Table 3. Virus-specific diagnostic codes (J12.3 or J21.1) were present either as the primary or a secondary diagnostic code in 173 (28%) laboratory-confirmed hMPV RTI hospitalizations.

**Table 1.** Demographic Characteristics of All-cause Respiratory Tract Infections and Laboratory-confirmed Human Metapneumovirus Respiratory Tract Infections Associated Hospital Admissions in Older Adults in Scotland (July 1, 2017–June 30, 2023)

		All-cause RTI (*n* = 231 508)	HMPV (*n* = 626)
Age	60–74 y	86 896 (37.53%)	269 (42.97%)
	≥75 y	144 612 (62.47%)	357 (57.03%)
Sex	Female	118 080 (51.00%)	343 (54.79%)
	Male	113 428 (49.00%)	283 (45.21%)
Ethnicity	White	199 926 (86.36%)	529 (84.50%)
	Non-White	29 593 (12.78%)	95 (15.18%)
	Data unavailable	1989 (0.86%)	2 (0.32%)
SIMD^a^	1	59 839 (25.85%)	82 (13.10%)
	2	54 435 (23.51%)	126 (20.3%)
	3	46 114 (19.92%)	144 (23.00%)
	4	37 967 (16.40%)	136 (21.73%)
	5	32 378 (13.99%)	134 (21.41%)
	Data unavailable	775 (0.33%)	4 (0.64%)

The values in brackets indicate the percentage of the total cases reported in each column.

Abbreviations: RTI, respiratory tract infections; hMPV, human metapneumovirus; SIMD, Scottish Index of Multiple Deprivation.

^a^SIMD ranks data zones in Scotland in order of deprivation. The data are presented as SIMD quintiles, with 1 indicating the most deprived area and higher quintiles progressively representing the less deprived areas.

The annual laboratory-confirmed and extrapolated hospital incidence of hMPV-associated RTIs in older adults in Scotland is presented in Table 2. Extrapolation was not possible for the 2020/21 season as Lothian did not report any hMPV cases in older adults during this season. We estimated 629 extrapolated hMPV-associated RTI hospitalization episodes in the 60–74 y age band, which was ∼2.3 times higher than the 269 laboratory-confirmed episodes. Similarly, we estimated 800 extrapolated hMPV-associated RTI hospitalization episodes in the ≥75 y age band, which was ∼2.2 times higher than the 357 laboratory-confirmed episodes. The extrapolated estimates were higher than the laboratory-confirmed estimates during all seasons (where extrapolation was possible) and were higher in the ≥75 y than the 60–74 y.

**Table 2.** Laboratory-confirmed and Extrapolated Annual Hospital Incidence of hMPV-associated RTIs (per 100 000) With 95% CI in Older Adults in Scotland by Age Bands During 6 Annual Seasons (2017–2023)^a^

Season	Laboratory-confirmed Annual Hospital Incidence in 60–74 y	Extrapolated Annual Hospital Incidence in 60–74 y	Laboratory-confirmed Annual Hospital Incidence in ≥75 y	Extrapolated Annual Hospital Incidence in ≥75 y	Laboratory-confirmed Annual Hospital Incidence in ≥60 y	Extrapolated Annual Hospital Incidence in ≥60 y
2017/18	8.7 (6.9–10.7)	11.4 (9.2–13.7)	26.9 (22.3–31.7)	42.6 (36.8–48.5)	14.8 (12.8–16.8)	21.7 (19.4–24.2)
2018/19	8.7 (7.0–10.5)	32.1 (28.8–35.7)	22.0 (17.6–26.5)	84.4 (76.2–93.2)	13.1 (11.2–15.0)	49.5 (46.0–53.3)
2019/20	5.5 (4.0–6.8)	13.1 (10.8–15.5)	15.6 (11.9–19.0)	27.6 (22.8–32.2)	8.8 (7.3–10.3)	17.9 (15.7–20.1)
2020/21	0.6 (0.2–1.1)	^b^	2.1 (0.8–3.6)	^b^	1.1 (0.6–1.7)	^b^
2021/22	3.1 (2–4.2)	7.1 (5.5–8.7)	5.6 (3.6–7.8)	9.7 (7.1–12.5)	3.9 (2.9–5.0)	8.0 (6.7–9.4)
2022/23	2.0 (1.1–3.0)	2.7 (1.7–3.8)	4.3 (2.5–6.2)	5.3 (3.3–7.4)	2.8 (2.0–3.7)	3.6 (2.6–4.5)

Abbreviation: hMPV, human metapneumovirus.

^a^Raw numbers not reported, as the presence of cells with counts <5 precluded their disclosure under data protection requirements.

^b^There were no lab-confirmed episodes in older adults in Lothian, and as a result, extrapolation was not possible.

The hMPV-associated RTI annual extrapolated hospital incidence, along with the all-cause RTI hospital incidence, is shown in Supplementary Table 4. Estimates for Lothian and other health boards are provided separately in Supplementary Table 5. The monthly laboratory-confirmed proportion positives in RTI in Lothian used for extrapolation are attached in Supplementary Table 6.

The sensitivity analyses conducted by limiting episodes with a specimen collected for testing within 3 days before to 3 days after the date of admission in Lothian demonstrated that the estimates were similar to the main analysis (where a 7-day cutoff postadmission was applied). These results are reported in Supplementary Table 7.

Over the 6 annual seasons in Scotland, 14%, 10%, 3%, and 11% laboratory-confirmed hMPV-associated RTI hospitalization episodes were associated with long hospital LOS, ICU admission, in-hospital death, and postdischarge death (up to 90 days postdischarge), respectively, in older adults (Table 3).

**Table 3.** Severity of Disease and Outcomes Associated With Laboratory-confirmed hMPV RTI Hospital Episodes in Older Adults in Scotland According to Age Bands and Region

	Age Band	Lothian	Other Health Boards	Scotland
Hospital LOS >5 d^a^	60–74 y	9.89 (4.40–16.48)	16.85 (11.80–22.47)	14.50 (10.78–18.59)
	≥75 y	14.16 (7.96–21.24)	14.34 (10.25–18.85)	14.29 (10.92–18.21)
	≥60 y	12.25 (8.32–17.26)	15.40 (12.09–18.73)	14.38 (11.66–17.25)
ICU admission rate (%)	60–74 y	7.69 (2.20–13.21)	19.10 (13.48–25.28)	15.24 (11.15–19.71)
	≥75 y	3.54 (0.88–7.08)	6.97 (4.09–9.85)	5.88 (3.64–8.12)
	≥60 y	5.39 (2.45–8.82)	12.09 (9.24–15.40)	9.90 (7.66–12.14)
In-hospital CFR (%)	60–74 y	1.10 (0.00–4.40)	1.69 (0.00–3.93)	1.49 (0.37–2.97)
	≥75 y	0.88 (0.00–2.65)	6.15 (3.28–9.43)	4.48 (2.52–6.72)
	≥60 y	0.98 (0.00–2.45)	4.27 (2.37- 6.16)	3.19 (1.92–4.63)
Postdischarge (90-d) CFR (%)	60–74 y	7.69 (2.20–13.19)	8.99 (5.06–12.92)	8.55 (5.58–12.27)
	≥75 y	10.62 (5.31–16.81)	13.93 (9.84–18.44)	12.89 (9.52–16.25)
	≥60 y	9.31 (5.39–13.24)	11.85 (9.00–14.93)	11.02 (8.63–13.58)

Abbreviations: hMPV, human metapneumovirus; LOS, length of stay; ICU, intensive care unit; CFR, case fatality rate.

^a^Estimates indicate the percentage of total cases requiring >5 d length of hospital stay along with 95% CI.

During the study period, there were 1841 laboratory-confirmed RSV and 7408 laboratory-confirmed Influenza A RTIs unique hospitalizations in older adults in Scotland. A virus-specific diagnostic code, either as the primary or a secondary diagnostic code, was present in 1160 (63%) of laboratory-confirmed RSV and 6815 (92%) of laboratory-confirmed Influenza A. The RSV and Influenza-specific codes are provided in Supplementary Table 2. The demographic characteristics of the laboratory-confirmed RSV and Influenza A episodes are presented in Supplementary Table 8. The hospital incidence point estimates were highest for Influenza A, followed by RSV and hMPV. These trends were similar across age bands, in Lothian and other health boards, and for both laboratory-confirmed and extrapolated estimates (Supplementary Tables 4 and 5). The proportion of episodes requiring long LOS, ICU admission rates, the in-hospital CFR, and postdischarge CFR estimates did not show consistent patterns across viruses, and their 95% confidence intervals generally overlapped (Supplementary Table 9).

## DISCUSSION

According to our current knowledge, this is the first study estimating the hMPV RTI hospital burden in older adults in Scotland. hMPV hospital incidence in older adults in Scotland increased with age, with the highest burden in ≥75 y, reaching up to 84 per 100 000 persons (2018/19 season) compared with 32 per 100 000 persons in the 60–74 y during the same season. hMPV hospital incidence dropped during the COVID-19 pandemic. Like incidence, postdischarge deaths were more common in the ≥75 y than 60–74 y, but a reverse trend was observed for ICU admissions.

Only 28% laboratory-confirmed hMPV RTI hospitalizations had hMPV-specific diagnostic codes (J12.3 or J21.1). These patterns are consistent with our previous observation for RSV and indicate that reliance on clinically coded data alone substantially underestimates the true hMPV burden [22]. Moreover, the observed variation in coding practices across the 3 respiratory viruses (63% for RSV and 92% for Influenza A) suggests a differential risk of bias in clinically coded records, underscoring the need to strengthen surveillance systems and improve diagnostic and coding accuracy to ensure valid comparisons. We postulate that these differences in coding practices could stem from diagnostic codes being assigned before the availability of laboratory confirmation, the absence of specific treatments or antivirals for hMPV, lower clinical recognition, and less well-defined care pathways for hMPV compared with influenza or RSV. However, further research is needed to explore and confirm these factors.

A published study from New Zealand reported hMPV-associated RTI hospital incidence of 44.3 and 92.0 per 100 000 in 65–79 y and ≥80 y, respectively, between 2012 and 2023 [23]. Our study used slightly different age bands (60–74 and ≥75 y) and examined individual seasons from 2017 to 2023; however, both studies observed a similar pattern of increasing incidence with age. While all our seasonal estimates were lower than the pooled estimate reported in the New Zealand study, the 2018/19 season estimates were closest, particularly in the ≥75 y in our study (84.4 per 100 000). This not only highlights year-on-year variations, but also differences in testing policies and hospitalization policies across regions. During the pre-COVID-19 seasons, observed yearly variations in hMPV hospital incidence may have resulted from shifts in the predominant genetic group within pre-existing lineages and a lack of immunity to the circulating strains [24]. Studies from other countries have also reported such year-on-year variations, supporting the presence of such patterns [25, 26]. The increasing hMPV incidence trend with increasing age in older adults was also consistent with the pattern observed in our systematic review [7]. Additionally, hMPV hospital incidence declined notably during the 2020/21 and 2021/22 seasons, reflecting reduced viral transmission due to COVID-19-related nonpharmaceutical interventions and possible under-reporting from disruptions in routine testing of other viruses, particularly during the early phase of the pandemic in 2020 (Table 2). This is also consistent with findings reported in the literature [23]. Notably, the hMPV hospital incidence remained low until the 2022/23 season, suggesting that hMPV activity continued to remain below prepandemic levels in older adults, and indicating the COVID-19 pandemic's continued impact on virus circulation (Table 2). As a result, it remains to be seen whether immunity debt related to the COVID-19 pandemic will contribute to increased hMPV incidence or associated severity in the upcoming seasons.

About 12% and 15% laboratory-confirmed hMPV hospital admissions in Lothian and other health boards, respectively, required long LOS (Table 3). It becomes important to identify the risk factors associated with long LOS to understand resource utilization and for effective planning in health systems. It must be noted that we included all inpatient episodes, including inpatient admissions lasting <1 day (*n* = 34 in Lothian and 76 in other health boards), as these cases, expectedly, required more resources than day cases. Despite the short hospital LOS, these episodes exert significant pressure over a short period on hospital bed capacity, particularly during the respiratory seasons. These impacts are unlikely to be captured in the routinely reported annual bed day statistics.

Our study reported higher ICU admission rates in hMPV-associated RTI admissions in 60–64 y than ≥75 y, possibly because invasive ICU treatments are often avoided for very old patients due to factors like age, frailty, pre-existing health status, and lower expected quality of life or survival benefit [27]. ICU admission rates during laboratory-confirmed hMPV hospitalizations seemed higher in other health boards (12%) than in Lothian (5%), likely reflecting selective testing for severe cases in other health boards and underlying population differences contributing to differential disease severity (Table 3). A recent U.S. study reported an ICU admission rate of ∼30% in the same age group, about 3 times higher than our estimate [28]. However, another study from Canada estimated an ICU admission rate of 11% in adults aged ≥50 y, which is comparable to our estimate of 10% in ≥60 y [29]. These comparisons again highlight the impact of differences in health systems, testing policies, ICU admission policies, and underlying populations across regions, likely affecting the estimates.

We estimated an in-hospital CFR of about 3% in laboratory-confirmed hMPV-associated RTI hospitalization episodes in older adults in our study. This is broadly similar to the 5% reported in the USA study for the same age group and the 2% reported in the Canadian study for ≥50 y [28, 29]. Similar to ICU admission rates, the in-hospital CFR of laboratory-confirmed hMPV RTIs appeared higher in other health boards (4%) than in Lothian (1%), with the difference particularly pronounced in the ≥75 y (6% vs 1%) (Table 3). This pattern supports the possibility that selective testing of more severe cases in health boards other than Lothian, as well as underlying population differences, may have contributed to the observed differences in outcomes. We also examined the postdischarge CFR within 90 days along with the in-hospital CFR. This is particularly relevant to older adults, in whom viral RTIs are frequently associated with a decline in overall health following the resolution of the acute respiratory episode [30, 31]. The postdischarge CFRs were higher than the in-hospital CFRs, confirming the extension of mortality risk beyond the acute phase (Table 3). Although it may not be directly attributable to hMPV RTIs, it could be reflecting a broader health deterioration associated with the RTI episodes.

Any true differences between the burden of hMPV and RSV or Influenza A may have been obscured by unclear testing policies in health boards other than Lothian, small sample sizes in Lothian upon stratification by age bands and seasons, and the lack of information on influenza immunization status. Therefore, statistical testing of the incidence differences between the viruses was not performed. The relative incidence trends with RSV and Influenza A observed in our study is, however, consistent with the literature [32–34]. The overlapping 95% CI also indicated that substantial differences in the severity and outcomes of hMPV and RSV or Influenza A were unlikely, although statistical testing was not undertaken due to varying testing policies (Supplementary Table 9).

We acknowledge some limitations of this study. First, the underlying population differences in age, ethnicity and deprivation between Lothian and other health boards would have differentially impacted the all-cause RTI ascertainment. Our extrapolation accounted for age-related differences in RTI risk, but not for ethnicity or SIMD, as sample sizes were too small after stratification. Moreover, complex interactions between incidence, healthcare access, and health behaviors in each group complicate the prediction of the overall impact of these factors on RTI ascertainment, potentially leading to a residual bias. Additionally, if population differences within the RTI populations are associated with differential risks for hMPV RTIs, the demographic differences in the RTI populations of Lothian and other health boards would have further affected the accuracy of extrapolated hMPV hospital incidence (Supplementary Table 3).

Second, disproportionate geographic differences affecting rural and remote health boards may have led to unequal testing and healthcare access across the different health boards [35]. To address the unequal testing issue, we used Lothian as a benchmark, where all RTI admissions were advised testing for hMPV during the study period, to extrapolate the hospital incidence in other health boards. Healthcare access was likely to be better in Lothian compared with other health boards, with a higher density of hospitals within its catchment area, indicating a shorter distance to hospitals [36]. This partly explains the higher hospital incidence of all-cause RTI in Lothian compared with other health boards, despite Lothian having a healthier population demographic than most health boards (Supplementary Table 5) [37]. The combination of a healthier population in Lothian and relatively limited access to healthcare for all-cause RTIs in some other health boards has likely resulted in the underestimation of hMPV incidence in Scotland, even after extrapolation. Furthermore, well-recognized challenges associated with viral RTI testing in older adults (not limited to specific health boards) include difficulty in obtaining clinical specimens, delayed care seeking and diminished and short-duration viral shedding, which may have resulted in further underestimation [38, 39].

Third, RTI outbreaks involving respiratory pathogens (including hMPV) in health boards other than Lothian may have resulted in overestimates or underestimates, respectively, of hMPV incidence during those outbreaks. A reverse effect could be expected in Lothian, where local outbreaks could lead to opposite effects on the estimation. Lastly, extrapolation should ideally have been performed weekly; however, low weekly case counts necessitated aggregation of monthly hMPV-positive RTI proportions in Lothian. This would likely have obscured the weekly variations and either underestimated or overestimated the short-term fluctuations in hMPV incidence.

Moreover, the lack of information on testing policies in individual health boards, except for Lothian, makes it difficult to determine the direction of potential bias on disease severity outcomes (proportion of episodes with long LOS, the ICU admission rate, and CFRs). Selective testing of hospital episodes with more severe disease may have led to overestimation of the proportion of cases with prolonged LOS, the ICU admission rate, or CFRs. However, the threshold for testing may have differed across health boards, potentially modifying the effect. More broadly, incomplete testing could have resulted in under-ascertainment of both laboratory-confirmed hMPV RTI hospital episodes and the episodes associated with severe disease. Consequently, the overall impact of incomplete testing on LOS, ICU admission rate, and CFRs remains uncertain.

## CONCLUSIONS

hMPV RTIs pose a considerable hospital burden in older adults in Scotland. Our estimates are likely underestimates due to incomplete testing, and underlying population and healthcare access differences in health boards, even after extrapolation. hMPV showed considerable year-on-year variation, highlighting the need for ongoing surveillance over a longer period. Adults aged ≥75 y reported higher hMPV incidence than those aged 60–64 y, indicating increased associated morbidity in the very old. The COVID-19 pandemic observed a significant drop in the hMPV hospital incidence, and the virus did not seem to return to prepandemic levels in older adults until the 2022/23 season. Our study demonstrates that the clinical burden and severity of hMPV could be comparable to RSV in older adults, but investments in diagnostics and continued surveillance are essential to develop robust comparisons.

## Supplementary Material

ofag057_Supplementary_Data

## Supplementary Data

Supplementary materials are available at *Open Forum Infectious Diseases* online. Consisting of data provided by the authors to benefit the reader, the posted materials are not copyedited and are the sole responsibility of the authors, so questions or comments should be addressed to the corresponding author.
