# Supplementary material for: Human Metapneumovirus-associated Hospital Burden in Older Adults in Scotland: A Retrospective Analysis
Source: Open Forum Infect Dis. 2026 Feb 11;13(2):ofag057. doi: 10.1093/ofid/ofag057 (PMC12931557; doi:10.1093/ofid/ofag057)
Supplement: ofag057_Supplementary_Data [file ofag057_supplementary_data.docx]

Table of Contents

[Supplementary Table S1: Underlying population characteristics in Lothian and other Scottish health boards in 2022 1](#_Toc210809541)

[Supplementary Table S2: ICD-10 respiratory tract infection diagnostic codes 2](#_Toc210809542)

[Supplementary Figure S1: Data cleaning and linking procedure 3](#_Toc210809543)

[Supplementary Text S1: Statistical analysis methods for extrapolation of hospital incidence 4](#_Toc210809544)

[Supplementary Figure S2: Flow diagram describing the methods of estimating the laboratory-confirmed hospital incidence and extrapolated hospital incidence in Scotland 6](#_Toc210809545)

[Supplementary Table S3: Study population characteristics by health boards – Lothian and other Scottish health boards 8](#_Toc210809546)

[Supplementary Table S4: Laboratory-confirmed and extrapolated hospital incidence per 100,000 persons (with 95% CI) of hMPV, RSV and Influenza A by age bands and season in older adults in Scotland 9](#_Toc210809547)

[Supplementary Table S5: Laboratory-confirmed and extrapolated hospital incidence per 100,000 persons (with 95% CI) of hMPV, RSV and Influenza A by age bands, season and health board (Lothian and other health boards) in older adults in Scotland 10](#_Toc210809548)

[Supplementary Table S6: Laboratory confirmed virus proportion positive in RTI admissions in Lothian by age bands and month 13](#_Toc210809549)

[Supplementary Table S7: Findings of sensitivity analysis conducted by limiting to episodes with specimen collected for viral testing between 3 days before and 3 days after the date of admission in older adults in Lothian by age bands and seasons 19](#_Toc210809550)

[Supplementary Table S8: Characteristics of all-cause RTI, hMPV, RSV, and Influenza A hospital episodes in older adults in Scotland (2017-2023) 20](#_Toc210809551)

[Supplementary Table S9: Severity and outcomes of hMPV-, RSV-, and Influenza A-associated RTI hospitalisations in older adults in Lothian and other health boards by age bands 21](#_Toc210809552)

# Supplementary Table S1: Underlying population characteristics in Lothian and other Scottish health boards in 2022 ^[[1]](#footnote-1)^^[[2]](#footnote-2)^

|  | **Lothian** | **Other Scottish health boards** | **Scotland** |
| --- | --- | --- | --- |
| **Total population (all ages)** | 904,628 | 4,535,214 | 5,439,842 |
| **Older adults (≥60y)** | 209,484  (23.16%) | 1,252,840  (27.62%) | 1,462,324  (26.88%) |
| **White ethnicity** | 809,550  (89.49%) | 4,242,323  (93.5%) | 5,051,873  (92.87%) |
| **Percentage of health board population in the 10% most deprived areas (SIMD 2020) ^[[3]](#footnote-3)^** | 4.2% | -- | 10% |

# Supplementary Table S2: ICD-10 respiratory tract infection diagnostic codes

| Condition | ICD-10 codes |
| --- | --- |
| Acute respiratory tract infections | J00\|J02\|J020\|J028\|J029\|J03\|J038\|J039\|J04\|J040\|J041\|J042\|J05\|J050\|J051\|J06\|J060\|J068\|J069\|J00\|J02\|J02.0\|J02.8\|J02.9\|J03\|J03.8\|J03.9\|J04\|J04.0\|J04.1\|J04.2\|J05\|J05.0\|J05.1\|J06\|J06.0\|J06.8\|J06.9 |
| Pneumonia and Influenza codes | J110\|J111\|J12\|J120\|J121\|J12\|J128\|J129\|J178\|J18\|J180\|J181\|J182\|J188\|J189\|J11.0\|J11.1\|J12\|J12.0\|J12.1\|J12\|J12.8\|J12.9\|J17.8\|J18\|J18.0\|J18.1\|J18.2\|J18.8\|J18.9 |
| Bronchiolitis and bronchitis | J20\|J200\|J201\|J202\|J206\|J207\|J208\|J209\|J21\|J218\|J219\|J40\|J20\|J20.0\|J20.1\|J20.2\|J20.6\|J20.7\|J20.8\|J20.9\|J21\|J21.8\|J21.9\|J40 |
| Unspecified LRTI | J22 |
| Human metapneumovirus codes | J123\|J211\|J12.3\|J21.1 |
| Respiratory syncytial virus codes | J121\|J205\|J210\|B974\|J12.1\|J20.5\|J21.0\|B97.4 |
| Flu codes | J09\|J10\|J100\|J101\|J108\|J11\|J11.8\|J09\|J10\|J10.0\|J10.1\|J10.8\|J11\|J11.8 |
| SARS-CoV-2 | U071\|U072\|U089\|U099\|U109\|U07.1\|U07.2\|U08.9\|U09.9\|U10.9 |

# Supplementary Figure S1: Data cleaning and linking procedure^[[4]](#footnote-4)^


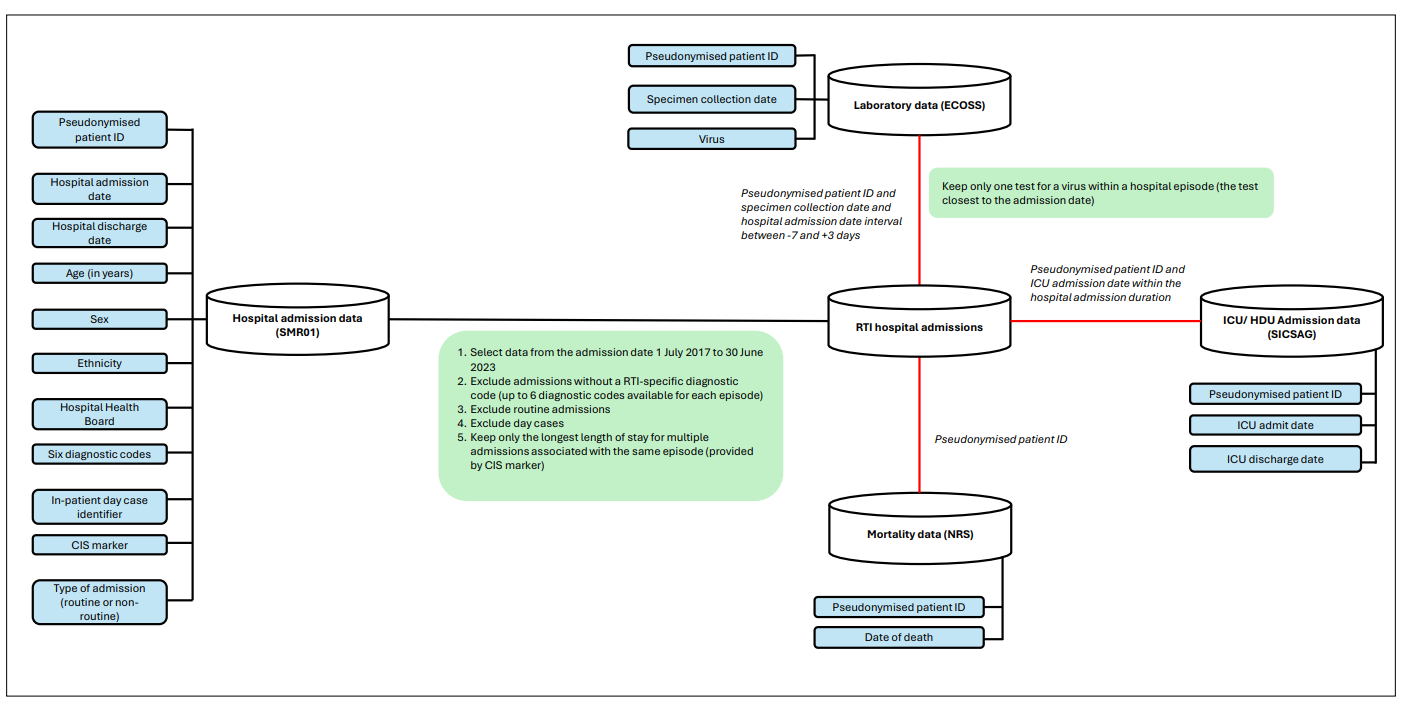


Keep only one test for a virus within a hospital episode (the test closest to the admission date)

# Supplementary Text S1: Statistical analysis methods for extrapolation of hospital incidence

The detailed steps are described below:

1. Calculate the hMPV proportion positive among all RTI admissions in Lothian by age bands (60-74y and ≥75y) and month of admission during each annual season.

$$Lothian monthly hMPV in RTI proportion positive_{\left\{ ai, si, mi \right\}}=\frac{\{Lothian lab confirmed hMPV_{\left\{ ai,si, mi \right\}}\}}{\{Lothian all RTI hospital admissions_{\left\{ ai,si, mi \right\}}\}}$$

(where $a$ is the age band, $s$ is the annual season and $m$ is the month of admission)

1. Apply the Lothian hMPV proportion positive in RTI to the corresponding RTI admissions in other health boards to obtain the extrapolated hMPV-associated RTI admissions in other health boards by age band and month of admission during each annual season.

$${RoS extrapolated monthly hMPV RTI admissions}_{\left\{ ai, si,mi \right\}}=\left\{ Lothian monthly hMPV in RTI proportion positive_{\left\{ ai,si,mi \right\}} \right\} \left\{ RoS all RTI admissions_{\left\{ ai,si,mi \right\}} \right\}$$

(where $a$ is the age band, $s$ is the annual season and $m$ is the month of admission; RoS stands for rest of Scotland i.e., health boards except Lothian)

1. Sum up the monthly hMPV-associated RTI admissions in Lothian and other health boards to calculate the annual (July – June) hMPV-associated RTI admissions in Scotland according to age band.

$${Extrapolated annual hMPV RTI admissions in Scotland}_{\left\{ ai, si \right\}}=\sum_{m=July yi}^{June y(i+1)} \left( {Lothian lab confirmed hMPV}_{\left\{ ai, mi \right\}}+{RoS extrapolated monthly hMPV RTI admissions}_{\left\{ ai, mi \right\}} \right)$$

(where $a$ is the age band, $s$ is the annual season and $m$ is the month of admission; RoS stands for rest of Scotland i.e., health boards except Lothian)

1. Estimate the extrapolated annual hMPV-associated RTI hospital incidence in Scotland by age band.

$$Extrapolated annual hMPV RTI incidence_{\left\{ ai, si \right\}}= \frac{\left\{ Extrapolated annual hMP{V RTI admissions}_{\left\{ ai, si \right\}} \right\}\times100,000}{\{{Scottish population}_{\left\{ ai, si \right\}}\}}$$

(where $a$ is the age band and $s$ is the annual season)

1. Estimate the 95% confidence interval using non-parametric bootstrap resampling (1,000 replicates) based on grouped count (age band and season) and population data.

# Supplementary Figure S2: Flow diagram describing the methods of estimating the laboratory-confirmed hospital incidence and extrapolated hospital incidence in Scotland

Laboratory data (ECOSS)

Hospital admission data (SMR01)

Population data by age band and year

All-cause RTI admissions in Scotland

n (Laboratory- confirmed hMPV-associated RTI hospital admissions in Scotland)

hMPV laboratory positive test results

n (Laboratory- confirmed annual hMPV-associated RTI hospital admissions in Scotland by age band and season)

Laboratory- confirmed annual hMPV-associated RTI hospital incidence with 95% CI by age band

**(a) Hospital incidence for Scotland based on laboratory-confirmed data from all Scottish health boards (without extrapolation)**

Laboratory data (ECOSS)

Hospital admission data (SMR01)

All-cause RTI admissions in Scotland

Laboratory- confirmed hMPV-associated RTI hospital admissions in Scotland

n (All-cause RTI hospital admissions in health boards except Lothian)

n (All-cause RTI hospital admissions in Lothian)

n (Laboratory- confirmed hMPV-associated RTI hospital admissions in Lothian)

n (Laboratory- confirmed hMPV-associated RTI hospital admissions in health boards except Lothian)

Monthly hMPV-proportion positive in RTI hospital admissions in Lothian by age band

n (Extrapolated monthly hMPV-associated RTI hospital admissions in health boards except Lothian by age band)

Population data by age band and year

n (Extrapolated annual hMPV-associated RTI hospital admissions in Scotland by age band)

Extrapolated annual hMPV-associated RTI hospital incidence with 95% CI by age band

hMPV positive test results

**(b) Hospital incidence for Scotland based on laboratory-confirmed data from Lothian and RTI hospital admissions in Scotland with extrapolation to health boards except Lothian**

# Supplementary Table S3: Study population characteristics by health boards – Lothian and other Scottish health boards^[[5]](#footnote-5)^

|  |  | **All-cause RTI in Lothian**  **(n = 33,986)** | **All-cause RTI in other health boards**  **(n = 197,522)** | **Laboratory-confirmed hMPV in Lothian**  **(n = 204)** | **Laboratory-confirmed hMPV in other health boards**  **(n = 422)** | **Laboratory-confirmed RSV in Lothian**  **(n = 461)** | **Laboratory-confirmed RSV in other health boards**  **(n = 1,380)** | **Laboratory-confirmed Influenza A in Lothian**  **(n = 1,484)** | **Laboratory-confirmed Influenza A in other health boards**  **(n = 5,924)** |
| --- | --- | --- | --- | --- | --- | --- | --- | --- | --- |
| **Age ^[[6]](#footnote-6)^** | 60-74 y | 13,183  (38.79%) | 73,713 (37.32%) | 91 (44.61%) | 178 (42.18%) | 185 (40.13%) | 573  (41.52%) | 617 (41.58%) | 2,679  (45.22%) |
|  | ≥75 y | 20,803  (61.21%) | 123,809  (62.68%) | 113 (55.39%) | 244 (57.82%) | 276  (59.87%) | 807  (58.48%) | 867  (58.42%) | 3,245  (54.78%) |
| **Sex** | Female | 17,391  (51.17%) | 100,689  (50.98%) | 119 (58.33%) | 224 (53.08%) | 265  (57.48%) | 810  (58.70%) | 814  (54.85%) | 3,287  (55.49%) |
|  | Male | 16,595  (48.83%) | 96,833  (49.02%) | 85 (41.67%) | 198 (46.92%) | 196  (42.52%) | 570  (41.30%) | 670  (45.15%) | 2,637  (44.51%) |
| **Ethnicity ^[[7]](#footnote-7)^** | White | 27,253  (80.19%) | 172,673  (87.42%) | 174  (85.29%) | 355 (84.12%) | 380 (82.43%) | 1209  (87.61%) | 1,212  (81.67%) | 5,180  (87.44%) |
|  | Non-White | 6,732  (19.81%) | 22,861 (11.57%) | 30 (14.71%) | 65 (15.40%) | 81 (17.57%) | 159  (11.52%) | 272  (18.33%) | 670  (11.31%) |
|  | Data unavailable | 1  (0%) | 1,988  (1.01%) | 0  (0%) | 2  (0.47%) | 0  (0%) | 12  (0.87%) | 0  (0%) | 74  (1.25%) |
| **SIMD ^[[8]](#footnote-8)^** | 1 | 5172  (15.22%) | 54667  (27.68%) | 25 (12.25%) | 57 (13.51%) | 62 (13.45%) | 365  (26.45%) | 222 (14.96%) | 1658  (27.99%) |
|  | 2 | 9371  (27.57%) | 45064 (22.81%) | 43 (21.08%) | 83  (19.67%) | 122 (26.46%) | 279  (20.22%) | 411 (27.70%) | 1305 (22.03%) |
|  | 3 | 5862 (17.25%) | 40252 (20.38%) | 44 (21.57%) | 100  (23.70%) | 86 (18.66%) | 290  (21.01%) | 262  (17.65%) | 1138 (19.21%) |
|  | 4 | 5217 (15.35%) | 32750 (16.58%) | 29 (14.22%) | 107 (25.36%) | 67 (14.53%) | 285  (20.65%) | 208 (14.02%) | 1049 (17.71%) |
|  | 5 | 8300 (24.42%) | 24078 (12.19%) | 62 (30.39%) | 72 (17.06%) | 124 (26.90%) | 150  (10.87%) | 377 (25.40%) | 748 (12.63%) |
|  | Data unavailable | 64 (0.19%) | 711 (0.36%) | 1  (0.49%) | 3  (0.71%) | 0  (0%) | 11  (0.80%) | 4  (0.27%) | 26 (0.44%) |

RTI = respiratory tract infections, hMPV = Human metapneumovirus, RSV = Respiratory syncytial virus, SIMD = Scottish Index of Multiple Deprivation

# Supplementary Table S4: Laboratory-confirmed and extrapolated hospital incidence per 100,000 persons (with 95% CI) of hMPV, RSV and Influenza A by age bands and season in older adults in Scotland

| **Season** | **Age band** | **All-cause RTI hospital incidence** | **Lab-confirmed hMPV hospital incidence** | **Extrapolated hMPV hospital incidence** | **Lab-confirmed RSV hospital incidence** | **Extrapolated RSV hospital incidence** | **Lab-confirmed Influenza A hospital incidence** | **Extrapolated Influenza A hospital incidence** |
| --- | --- | --- | --- | --- | --- | --- | --- | --- |
| 2017/18 | 60-74y | 1682.9 (1656.9 - 1709.7) | 8.7 (6.9 - 10.7) | 11.3 (9.2 - 13.7) | 14.9 (12.3 - 17.4) | 20.4 (17.8 - 23.3) | 93.3 (86.8 - 99.6) | 117.8 (110.6 – 125.0) |
| 2018/19 | 60-74y | 1533.3 (1509.3 - 1556.7) | 8.7 (7.0 - 10.5) | 32.1 (28.8 - 35.7) | 17 (14.3 - 19.6) | 29.8 (26.4 - 33.3) | 74.1 (68.4 - 79.8) | 96.0 (90.1 - 102.3) |
| 2019/20 | 60-74y | 1487.8 (1463.4 - 1512.8) | 5.5 (4.0 - 6.8) | 13.1 (10.8 - 15.5) | 21 (18.2 - 23.9) | 22.1 (19.2 - 25.2) | 59.8 (55.3 - 64.8) | 70.5 (65.3 – 76.0) |
| 2020/21 | 60-74y | 1330.7 (1307.2 - 1352.6) | 0.6 (0.2 - 1.1) | ^[[9]](#footnote-9)^ | 0.1 (0 - 0.4) | * | 0.8 (0.3 - 1.4) | * |
| 2021/22 | 60-74y | 1702.4 (1677.3 - 1728.1) | 3.1 (2.0 - 4.2) | 7.1 (5.4 - 8.7) | 4.3 (3.1 - 5.7) | 6.8 (5.2 - 8.5) | 13.9 (11.5 – 16.0) | 15.5 (13.1 - 17.9) |
| 2022/23 | 60-74y | 1384.1 (1361.3 - 1406.5) | 2.0 (1.1 – 3.0) | 2.7 (1.7 - 3.8) | 22.6 (19.7 - 25.6) | 40.4 (36.8 - 44.5) | 105.6 (99.4 - 111.8) | 113.9 (107.3 - 120.8) |
| 2017/18 | 75+y | 6009.7 (5933.9 - 6075.8) | 26.9 (22.3 - 31.7) | 42.6 (36.8 - 48.5) | 36.8 (31.3 - 42.3) | 67.7 (60.2 - 74.9) | 318.2 (301.5 - 334.1) | 486.4 (466.5 - 504.7) |
| 2018/19 | 75+y | 5095.4 (5026.1 - 5157.8) | 22.0 (17.6 - 26.5) | 84.4 (76.2 - 93.2) | 49.7 (43.2 - 56.2) | 111.2 (101.3 - 120.5) | 102.0 (93.2 - 111.2) | 182.6 (171.3 - 194.9) |
| 2019/20 | 75+y | 5192.2 (5128.2 - 5252.2) | 15.6 (11.9 – 19.0) | 27.6 (22.8 - 32.2) | 59.7 (53.1 - 67.2) | 91.4 (83.2 - 100.3) | 152.8 (142.3 - 164.7) | 264.8 (249.4 - 279.7) |
| 2020/21 | 75+y | 4223.8 (4170.3 - 4278.3) | 2.1 (0.8 - 3.6) | * | 0.4 (0 - 1) | * | 1.3 (0.4 - 2.3) | * |
| 2021/22 | 75+y | 5150.5 (5090.1 - 5208) | 5.6 (3.6 - 7.8) | 9.7 (7 - 12.4) | 9 (6.4 - 11.8) | 21.1 (17.1 - 25.1) | 36.4 (31.1 - 41.8) | 46.8 (41.0 - 52.4) |
| 2022/23 | 75+y | 4588.9 (4526.1 - 4644.9) | 4.3 (2.5 - 6.2) | 5.3 (3.3 - 7.4) | 69.8 (62.4 - 77) | 100.1 (91.1 - 108.6) | 251.8 (237.6 - 265.5) | 271.7 (257.8 – 285.0) |
| 2017/18 | 60+y | 3117.2 (3090 - 3146.8) | 14.8 (12.8 - 16.8) | 21.7 (19.4 - 24.2) | 22.1 (19.6 - 24.5) | 36.1 (33.0 - 39.3) | 167.9 (161.4 – 175.0) | 240.0 (231.9 - 248.4) |
| 2018/19 | 60+y | 2718.5 (2691 - 2747.2) | 13.1 (11.2 – 15.0) | 49.5 (46.0 - 53.3) | 27.9 (25 - 30.5) | 56.9 (52.8 - 60.8) | 83.4 (78.5 - 88.1) | 124.8 (118.8 - 130.7) |
| 2019/20 | 60+y | 2711.7 (2685.4 - 2738) | 8.8 (7.3 - 10.3) | 17.9 (15.7 - 20.1) | 33.8 (30.7 - 36.9) | 45.0 (41.4 - 48.3) | 90.5 (85.6 - 95.6) | 134.7 (129.3 - 141.1) |
| 2020/21 | 60+y | 2286.1 (2261.3 - 2309.8) | 1.1 (0.6 - 1.7) | * | 0.2 (0 - 0.5) | * | 1.0 (0.5 - 1.5) | * |
| 2021/22 | 60+y | 2869.5 (2842.9 - 2895.4) | 3.9 (2.9 – 5.0) | 8.0 (6.7 - 9.4) | 5.9 (4.8 - 7.3) | 11.6 (9.9 - 13.3) | 21.5 (19.2 - 23.9) | 26.1 (23.4 - 28.8) |
| 2022/23 | 60+y | 2481.6 (2457.9 - 2506.3) | 2.8 (2.0 - 3.7) | 3.6 (2.6 - 4.5) | 38.7 (35.5 - 41.9) | 60.9 (56.9 - 64.7) | 155.7 (149.3 - 162) | 167.9 (161.1 - 175.1) |

# Supplementary Table S5: Laboratory-confirmed and extrapolated hospital incidence per 100,000 persons (with 95% CI) of hMPV, RSV and Influenza A by age bands, season and health board (Lothian and other health boards) in older adults in Scotland

| **Season** | **Age band** | **Region** | **All-cause RTI hospital incidence** | **Lab-confirmed hMPV hospital incidence** | **Extrapolated hMPV hospital incidence** | **Lab-confirmed RSV hospital incidence** | **Extrapolated RSV hospital incidence** | **Lab-confirmed Influenza A hospital incidence** | **Extrapolated Influenza A hospital incidence** |
| --- | --- | --- | --- | --- | --- | --- | --- | --- | --- |
| 2017/18 | 60-74y | Lothian | 1850.9 (1777.9 - 1927.8) | 11.5 (6.2 - 18.5) | NA (NA - NA) | 20.8 (13.1 - 29.2) | NA (NA - NA) | 129.1 (109.9 - 149.1) | NA (NA - NA) |
| 2017/18 | 60-74y | Other health boards | 1655.1 (1624.5 - 1681.9) | 8.3 (6.4 - 10.5) | 11.3 (8.9 - 13.5) | 13.9 (11.3 - 16.6) | 20.4 (17.2 - 23.6) | 87.4 (81.1 - 93.8) | 115.9 (108.5 - 123.7) |
| 2018/19 | 60-74y | Lothian | 1688.3 (1621.4 - 1755.3) | 36.1 (26.3 - 46.7) | NA (NA - NA) | 36.1 (26.3 - 46.7) | NA (NA - NA) | 103.1 (85.8 - 119.7) | NA (NA - NA) |
| 2018/19 | 60-74y | Other health boards | 1507.5 (1479.9 - 1534.3) | 4.1 (2.8 - 5.5) | 31.5 (27.5 - 35.2) | 13.8 (11.5 - 16.3) | 28.7 (25.2 - 32.4) | 69.3 (63.7 - 75.1) | 94.9 (88.6 - 101.5) |
| 2019/20 | 60-74y | Lothian | 1641.8 (1577.0 - 1712.5) | 12.5 (7.4 - 18.4) | NA (NA - NA) | 25.8 (17.7 - 34.6) | NA (NA - NA) | 79.6 (64.8 - 95.8) | NA (NA - NA) |
| 2019/20 | 60-74y | Other health boards | 1462.2 (1434.5 – 1488.0) | 4.3 (3.1 - 5.8) | 13.2 (10.7 - 15.8) | 20.1 (17.0 - 23.3) | 21.5 (18.3 - 24.9) | 56.5 (51.6 - 61.5) | 69.0 (63.1 - 74.8) |
| 2020/21 | 60-74y | Lothian | 1352.2 (1290.8 - 1411.5) | ^[[10]](#footnote-10)^ | NA (NA - NA) | * | NA (NA - NA) | * | NA (NA - NA) |
| 2020/21 | 60-74y | Other health boards | 1327.1 (1303.2 - 1350.4) | 0.7 (0.2 - 1.3) | ^[[11]](#footnote-11)^ | 0.1 (0 - 0.4) | † | 1.0 (0.4 - 1.7) | † |
| 2021/22 | 60-74y | Lothian | 1737.3 (1674.0 - 1807.8) | 6.5 (2.9 - 11.5) | NA (NA - NA) | 7.2 (3.6 - 12.2) | NA (NA - NA) | 14.4 (8.6 - 20.2) | NA (NA - NA) |
| 2021/22 | 60-74y | Other health boards | 1696.6 (1670.4 - 1722.2) | 2.5 (1.4 - 3.6) | 7.2 (5.5 - 8.9) | 3.8 (2.6 - 5.3) | 6.7 (5.0 - 8.4) | 13.8 (11.5 - 16.3) | 15.7 (13.0 - 18.3) |
| 2022/23 | 60-74y | Lothian | 1430.7 (1371.1 - 1492.6) | 1.4 (0 - 3.6) | NA (NA - NA) | 46.2 (35.5 - 58.2) | NA (NA - NA) | 130.7 (113.6 - 149.1) | NA (NA - NA) |
| 2022/23 | 60-74y | Other health boards | 1376.3 (1349.7 - 1401.1) | 2.1 (1.3 - 3.2) | 2.9 (1.8 – 4.0) | 18.6 (15.6 - 21.5) | 39.5 (35.1 - 43.9) | 101.5 (94.7 – 108.0) | 111.1 (104.0 - 118.3) |
| 2017/18 | ≥75y | Lothian | 6376.7 (6188.2 - 6565.2) | 43.2 (29.3 - 60.2) | NA (NA - NA) | 74.1 (52.5 - 95.8) | NA (NA - NA) | 526.6 (474.1 - 579.1) | NA (NA - NA) |
| 2017/18 | ≥75y | Other health boards | 5948.6 (5874.3 - 6021.6) | 24.2 (19.3 - 29.1) | 42.5 (36.0 - 49.4) | 30.6 (25.2 - 36.5) | 66.7 (58.9 - 74.8) | 283.5 (268.1 – 300.0) | 479.7 (458.8 - 502.6) |
| 2018/19 | ≥75y | Lothian | 5220.5 (5074.1 - 5396.9) | 92.0 (70.9 - 114.7) | NA (NA - NA) | 123.7 (98.0 - 150.9) | NA (NA - NA) | 191.6 (159.9 - 223.2) | NA (NA - NA) |
| 2018/19 | ≥75y | Other health boards | 5074.5 (5001.5 - 5141.1) | 10.3 (7.3 - 13.3) | 83.2 (74.5 - 92.1) | 37.4 (31.6 - 43.9) | 109.1 (98.6 - 119.5) | 87.1 (78.1 - 96.6) | 181.1 (167.4 - 193.5) |
| 2019/20 | ≥75y | Lothian | 5343.8 (5176.3 - 5511.3) | 26.9 (15.0 - 38.9) | NA (NA - NA) | 94.2 (71.8 - 118.1) | NA (NA - NA) | 273.6 (236.2 - 312.5) | NA (NA - NA) |
| 2019/20 | ≥75y | Other health boards | 5167.0 (5100.3 - 5231.7) | 13.7 (10.2 - 17.4) | 27.7 (23.2 - 32.6) | 54.0 (46.8 - 61.5) | 91.0 (82.4 - 100.1) | 132.7 (121.2 - 143.6) | 263.3 (248.1 - 278.5) |
| 2020/21 | ≥75y | Lothian | 4154.7 (4017.4 - 4308.1) | * | NA (NA - NA) | * | NA (NA - NA) | * | NA (NA - NA) |
| 2020/21 | ≥75y | Other health boards | 4235.4 (4176.9 - 4297.8) | 2.5 (1.0 - 3.9) | † | 0.5 (0 - 1.2) | † | 1.5 (0.5 - 2.7) | † |
| 2021/22 | ≥75y | Lothian | 4992.3 (4830.6 - 5147.1) | 5.6 (1.4 - 11.2) | NA (NA - NA) | 20.9 (11.2 - 32.1) | NA (NA - NA) | 40.4 (26.5 - 55.7) | NA (NA - NA) |
| 2021/22 | ≥75y | Other health boards | 5177.2 (5112.8 - 5244.1) | 5.6 (3.5 – 8.0) | 10.4 (7.5 - 13.1) | 7.0 (4.7 - 9.6) | 21.2 (16.7 - 25.6) | 35.7 (30.3 - 41.3) | 47.9 (41.5 - 54.7) |
| 2022/23 | ≥75y | Lothian | 4333.9 (4185.4 - 4471.6) | 2.7 (0 - 6.8) | NA (NA - NA) | 91.8 (70.2 - 113.4) | NA (NA - NA) | 252.4 (217.3 - 290.2) | NA (NA - NA) |
| 2022/23 | ≥75y | Other health boards | 4632.0 (4566.3 - 4692.9) | 4.6 (2.7 - 6.8) | 5.7 (3.7 – 8.0) | 66.1 (58.6 - 74.6) | 101.6 (92.1 – 111.0) | 251.7 (235.7 - 266.7) | 274.9 (260.6 - 290.9) |
| 2017/18 | ≥60y | Lothian | 3354.9 (3269.7 - 3433.4) | 22.1 (15.9 - 29.3) | NA (NA - NA) | 38.5 (29.3 - 47.7) | NA (NA - NA) | 261.2 (239.2 - 282.8) | NA (NA - NA) |
| 2017/18 | ≥60y | Other health boards | 3077.7 (3048.2 - 3109.1) | 13.5 (11.5 - 15.7) | 21.6 (19.0 - 24.3) | 19.4 (17.1 - 22.2) | 35.7 (32.5 - 39.2) | 152.4 (145.3 - 159.3) | 236.5 (227.8 - 245.7) |
| 2018/19 | ≥60y | Lothian | 2863.8 (2790.0 - 2939.1) | 54.7 (44.7 - 65.3) | NA (NA - NA) | 65.3 (54.2 - 77.3) | NA (NA - NA) | 132.5 (117.0 - 148.1) | NA (NA - NA) |
| 2018/19 | ≥60y | Other health boards | 2694.3 (2666.3 – 2725.0) | 6.2 (4.8 - 7.6) | 48.7 (44.8 - 52.7) | 21.6 (18.9 - 24.3) | 55.5 (50.9 - 59.5) | 75.2 (70.1 - 80.3) | 123.6 (117.1 - 129.9) |
| 2019/20 | ≥60y | Lothian | 2863.6 (2791.1 - 2940.1) | 17.3 (12.3 - 23.2) | NA (NA - NA) | 48.4 (39.0 - 58.2) | NA (NA - NA) | 143.6 (126.3 - 159.4) | NA (NA - NA) |
| 2019/20 | ≥60y | Other health boards | 2686.4 (2656.8 - 2715.9) | 7.4 (5.9 – 9.0) | 18.0 (15.5 - 20.4) | 31.3 (28.4 - 34.5) | 44.4 (40.8 - 48.1) | 81.7 (76.6 - 86.9) | 133.2 (127.2 – 140.0) |
| 2020/21 | ≥60y | Lothian | 2280.0 (2216.1 - 2343.4) | * | NA (NA - NA) | * | NA (NA - NA) | * | NA (NA - NA) |
| 2020/21 | ≥60y | Other health boards | 2287.1 (2260.1 - 2314.3) | 1.3 (0.7 – 2.0) | † | 0.2 (0 - 0.6) | † | 1.1 (0.6 - 1.8) | † |
| 2021/22 | ≥60y | Lothian | 2845.3 (2775.1 - 2920.8) | 6.2 (3.3 – 10.0) | NA (NA - NA) | 11.9 (7.6 - 17.1) | NA (NA - NA) | 23.2 (17.1 - 29.9) | NA (NA - NA) |
| 2021/22 | ≥60y | Other health boards | 2873.5 (2844.6 - 2902.3) | 3.6 (2.5 - 4.6) | 8.3 (6.7 - 9.8) | 4.9 (3.7 - 6.2) | 11.6 (9.8 - 13.3) | 21.2 (18.7 - 23.8) | 26.6 (23.6 - 29.4) |
| 2022/23 | ≥60y | Lothian | 2431.5 (2363.1 - 2495.7) | 1.9 (0.5 - 4.2) | NA (NA - NA) | 61.9 (51.2 - 72.6) | NA (NA - NA) | 172.6 (155.4 - 189.4) | NA (NA - NA) |
| 2022/23 | ≥60y | Other health boards | 2490.0 (2462.2 - 2515.3) | 3.0 (2 - 3.9) | 3.9 (2.7 – 5.0) | 34.9 (31.7 – 38.0) | 60.7 (56.2 - 64.7) | 152.9 (145.9 - 159.5) | 167.2 (160.0 - 174.5) |

RTI = respiratory tract infections, hMPV = Human metapneumovirus, RSV = Respiratory syncytial virus

# Supplementary Table S6: Laboratory confirmed virus proportion positive in RTI admissions in Lothian by age bands and month

| **Virus** | **Age band** | **Virus proportion positive in RTI episodes** | **Month and year of hospital admission** |
| --- | --- | --- | --- |
| hMPV | 60-74y | 0.006 | Aug 2017 |
| hMPV | 60-74y | 0.006 | Oct 2017 |
| hMPV | 60-74y | 0.006 | Dec 2017 |
| hMPV | 60-74y | 0.006 | Jan 2018 |
| hMPV | 60-74y | 0.018 | Feb 2018 |
| hMPV | 60-74y | 0.021 | Apr 2018 |
| hMPV | 60-74y | 0.006 | Jun 2018 |
| hMPV | 60-74y | 0.015 | Jul 2018 |
| hMPV | 60-74y | 0.017 | Nov 2018 |
| hMPV | 60-74y | 0.054 | Dec 2018 |
| hMPV | 60-74y | 0.046 | Jan 2019 |
| hMPV | 60-74y | 0.013 | Feb 2019 |
| hMPV | 60-74y | 0.035 | Mar 2019 |
| hMPV | 60-74y | 0.005 | Apr 2019 |
| hMPV | 60-74y | 0.012 | May 2019 |
| hMPV | 60-74y | 0.012 | Jun 2019 |
| hMPV | 60-74y | 0.026 | Jul 2019 |
| hMPV | 60-74y | 0.003 | Dec 2019 |
| hMPV | 60-74y | 0.045 | Jan 2020 |
| hMPV | 60-74y | 0.009 | Mar 2020 |
| hMPV | 60-74y | 0.009 | Oct 2021 |
| hMPV | 60-74y | 0.011 | Nov 2021 |
| hMPV | 60-74y | 0.018 | Dec 2021 |
| hMPV | 60-74y | 0.005 | Jan 2022 |
| hMPV | 60-74y | 0.006 | Sep 2022 |
| hMPV | 60-74y | 0.003 | Dec 2022 |
| hMPV | 75+y | 0.003 | Nov 2017 |
| hMPV | 75+y | 0.001 | Dec 2017 |
| hMPV | 75+y | 0.011 | Jan 2018 |
| hMPV | 75+y | 0.009 | Feb 2018 |
| hMPV | 75+y | 0.014 | Mar 2018 |
| hMPV | 75+y | 0.026 | Apr 2018 |
| hMPV | 75+y | 0.015 | May 2018 |
| hMPV | 75+y | 0.005 | Sep 2018 |
| hMPV | 75+y | 0.008 | Oct 2018 |
| hMPV | 75+y | 0.019 | Nov 2018 |
| hMPV | 75+y | 0.033 | Dec 2018 |
| hMPV | 75+y | 0.038 | Jan 2019 |
| hMPV | 75+y | 0.032 | Feb 2019 |
| hMPV | 75+y | 0.01 | Mar 2019 |
| hMPV | 75+y | 0.016 | Apr 2019 |
| hMPV | 75+y | 0.015 | Jun 2019 |
| hMPV | 75+y | 0.003 | Oct 2019 |
| hMPV | 75+y | 0.01 | Nov 2019 |
| hMPV | 75+y | 0.007 | Dec 2019 |
| hMPV | 75+y | 0.017 | Jan 2020 |
| hMPV | 75+y | 0.006 | Feb 2020 |
| hMPV | 75+y | 0.006 | Mar 2020 |
| hMPV | 75+y | 0.006 | Oct 2021 |
| hMPV | 75+y | 0.008 | Nov 2021 |
| hMPV | 75+y | 0.003 | Jan 2023 |
| hMPV | 75+y | 0.003 | Mar 2023 |
| RSV | 60-74y | 0.006 | Sep 2017 |
| RSV | 60-74y | 0.036 | Dec 2017 |
| RSV | 60-74y | 0.02 | Jan 2018 |
| RSV | 60-74y | 0.004 | Feb 2018 |
| RSV | 60-74y | 0.005 | Mar 2018 |
| RSV | 60-74y | 0.021 | Apr 2018 |
| RSV | 60-74y | 0.007 | May 2018 |
| RSV | 60-74y | 0.008 | Aug 2018 |
| RSV | 60-74y | 0.006 | Oct 2018 |
| RSV | 60-74y | 0.051 | Nov 2018 |
| RSV | 60-74y | 0.082 | Dec 2018 |
| RSV | 60-74y | 0.028 | Jan 2019 |
| RSV | 60-74y | 0.013 | Feb 2019 |
| RSV | 60-74y | 0.012 | Mar 2019 |
| RSV | 60-74y | 0.007 | Jul 2019 |
| RSV | 60-74y | 0.008 | Aug 2019 |
| RSV | 60-74y | 0.02 | Oct 2019 |
| RSV | 60-74y | 0.049 | Nov 2019 |
| RSV | 60-74y | 0.055 | Dec 2019 |
| RSV | 60-74y | 0.013 | Jan 2020 |
| RSV | 60-74y | 0.005 | Feb 2020 |
| RSV | 60-74y | 0.009 | Oct 2021 |
| RSV | 60-74y | 0.005 | Nov 2021 |
| RSV | 60-74y | 0.018 | Dec 2021 |
| RSV | 60-74y | 0.009 | Mar 2022 |
| RSV | 60-74y | 0.004 | Jun 2022 |
| RSV | 60-74y | 0.008 | Jul 2022 |
| RSV | 60-74y | 0.007 | Aug 2022 |
| RSV | 60-74y | 0.033 | Sep 2022 |
| RSV | 60-74y | 0.031 | Oct 2022 |
| RSV | 60-74y | 0.086 | Nov 2022 |
| RSV | 60-74y | 0.053 | Dec 2022 |
| RSV | 60-74y | 0.047 | Jan 2023 |
| RSV | 60-74y | 0.017 | Feb 2023 |
| RSV | 60-74y | 0.026 | Mar 2023 |
| RSV | 60-74y | 0.019 | Apr 2023 |
| RSV | 60-74y | 0.009 | May 2023 |
| RSV | 75+y | 0.004 | Sep 2017 |
| RSV | 75+y | 0.003 | Oct 2017 |
| RSV | 75+y | 0.018 | Nov 2017 |
| RSV | 75+y | 0.036 | Dec 2017 |
| RSV | 75+y | 0.013 | Jan 2018 |
| RSV | 75+y | 0.009 | Feb 2018 |
| RSV | 75+y | 0.005 | Mar 2018 |
| RSV | 75+y | 0.01 | Apr 2018 |
| RSV | 75+y | 0.005 | Aug 2018 |
| RSV | 75+y | 0.005 | Sep 2018 |
| RSV | 75+y | 0.011 | Oct 2018 |
| RSV | 75+y | 0.042 | Nov 2018 |
| RSV | 75+y | 0.069 | Dec 2018 |
| RSV | 75+y | 0.056 | Jan 2019 |
| RSV | 75+y | 0.019 | Feb 2019 |
| RSV | 75+y | 0.01 | Mar 2019 |
| RSV | 75+y | 0.003 | Apr 2019 |
| RSV | 75+y | 0.008 | Jun 2019 |
| RSV | 75+y | 0.004 | Sep 2019 |
| RSV | 75+y | 0.01 | Oct 2019 |
| RSV | 75+y | 0.04 | Nov 2019 |
| RSV | 75+y | 0.066 | Dec 2019 |
| RSV | 75+y | 0.036 | Jan 2020 |
| RSV | 75+y | 0.003 | Feb 2020 |
| RSV | 75+y | 0.003 | Mar 2020 |
| RSV | 75+y | 0.01 | Oct 2021 |
| RSV | 75+y | 0.004 | Nov 2021 |
| RSV | 75+y | 0.01 | Dec 2021 |
| RSV | 75+y | 0.014 | Jan 2022 |
| RSV | 75+y | 0.004 | Feb 2022 |
| RSV | 75+y | 0.006 | Mar 2022 |
| RSV | 75+y | 0.003 | Jun 2022 |
| RSV | 75+y | 0.003 | Jul 2022 |
| RSV | 75+y | 0.008 | Sep 2022 |
| RSV | 75+y | 0.022 | Oct 2022 |
| RSV | 75+y | 0.058 | Nov 2022 |
| RSV | 75+y | 0.057 | Dec 2022 |
| RSV | 75+y | 0.039 | Jan 2023 |
| RSV | 75+y | 0.015 | Feb 2023 |
| RSV | 75+y | 0.003 | Mar 2023 |
| RSV | 75+y | 0.016 | May 2023 |
| Influenza A | 60-74y | 0.006 | Aug 2017 |
| Influenza A | 60-74y | 0.006 | Sep 2017 |
| Influenza A | 60-74y | 0.006 | Oct 2017 |
| Influenza A | 60-74y | 0.031 | Nov 2017 |
| Influenza A | 60-74y | 0.218 | Dec 2017 |
| Influenza A | 60-74y | 0.175 | Jan 2018 |
| Influenza A | 60-74y | 0.053 | Feb 2018 |
| Influenza A | 60-74y | 0.039 | Mar 2018 |
| Influenza A | 60-74y | 0.011 | Apr 2018 |
| Influenza A | 60-74y | 0.007 | May 2018 |
| Influenza A | 60-74y | 0.018 | Jun 2018 |
| Influenza A | 60-74y | 0.008 | Aug 2018 |
| Influenza A | 60-74y | 0.006 | Oct 2018 |
| Influenza A | 60-74y | 0.061 | Dec 2018 |
| Influenza A | 60-74y | 0.164 | Jan 2019 |
| Influenza A | 60-74y | 0.2 | Feb 2019 |
| Influenza A | 60-74y | 0.081 | Mar 2019 |
| Influenza A | 60-74y | 0.041 | Apr 2019 |
| Influenza A | 60-74y | 0.012 | May 2019 |
| Influenza A | 60-74y | 0.012 | Jun 2019 |
| Influenza A | 60-74y | 0.007 | Jul 2019 |
| Influenza A | 60-74y | 0.016 | Aug 2019 |
| Influenza A | 60-74y | 0.014 | Sep 2019 |
| Influenza A | 60-74y | 0.015 | Oct 2019 |
| Influenza A | 60-74y | 0.044 | Nov 2019 |
| Influenza A | 60-74y | 0.224 | Dec 2019 |
| Influenza A | 60-74y | 0.099 | Jan 2020 |
| Influenza A | 60-74y | 0.019 | Feb 2020 |
| Influenza A | 60-74y | 0.004 | Mar 2020 |
| Influenza A | 60-74y | 0.009 | Dec 2021 |
| Influenza A | 60-74y | 0.006 | Feb 2022 |
| Influenza A | 60-74y | 0.026 | Mar 2022 |
| Influenza A | 60-74y | 0.053 | Apr 2022 |
| Influenza A | 60-74y | 0.006 | May 2022 |
| Influenza A | 60-74y | 0.004 | Jun 2022 |
| Influenza A | 60-74y | 0.004 | Jul 2022 |
| Influenza A | 60-74y | 0.013 | Aug 2022 |
| Influenza A | 60-74y | 0.033 | Sep 2022 |
| Influenza A | 60-74y | 0.037 | Oct 2022 |
| Influenza A | 60-74y | 0.107 | Nov 2022 |
| Influenza A | 60-74y | 0.38 | Dec 2022 |
| Influenza A | 60-74y | 0.165 | Jan 2023 |
| Influenza A | 60-74y | 0.017 | Feb 2023 |
| Influenza A | 60-74y | 0.009 | Apr 2023 |
| Influenza A | 60-74y | 0.017 | May 2023 |
| Influenza A | 75+y | 0.004 | Jul 2017 |
| Influenza A | 75+y | 0.004 | Aug 2017 |
| Influenza A | 75+y | 0.011 | Sep 2017 |
| Influenza A | 75+y | 0.014 | Oct 2017 |
| Influenza A | 75+y | 0.058 | Nov 2017 |
| Influenza A | 75+y | 0.258 | Dec 2017 |
| Influenza A | 75+y | 0.181 | Jan 2018 |
| Influenza A | 75+y | 0.038 | Feb 2018 |
| Influenza A | 75+y | 0.033 | Mar 2018 |
| Influenza A | 75+y | 0.023 | Apr 2018 |
| Influenza A | 75+y | 0.007 | May 2018 |
| Influenza A | 75+y | 0.008 | Oct 2018 |
| Influenza A | 75+y | 0.029 | Dec 2018 |
| Influenza A | 75+y | 0.101 | Jan 2019 |
| Influenza A | 75+y | 0.097 | Feb 2019 |
| Influenza A | 75+y | 0.071 | Mar 2019 |
| Influenza A | 75+y | 0.035 | Apr 2019 |
| Influenza A | 75+y | 0.023 | May 2019 |
| Influenza A | 75+y | 0.016 | Sep 2019 |
| Influenza A | 75+y | 0.01 | Oct 2019 |
| Influenza A | 75+y | 0.053 | Nov 2019 |
| Influenza A | 75+y | 0.198 | Dec 2019 |
| Influenza A | 75+y | 0.148 | Jan 2020 |
| Influenza A | 75+y | 0.019 | Feb 2020 |
| Influenza A | 75+y | 0.006 | Mar 2020 |
| Influenza A | 75+y | 0.003 | Dec 2021 |
| Influenza A | 75+y | 0.007 | Jan 2022 |
| Influenza A | 75+y | 0.008 | Feb 2022 |
| Influenza A | 75+y | 0.036 | Mar 2022 |
| Influenza A | 75+y | 0.025 | Apr 2022 |
| Influenza A | 75+y | 0.011 | May 2022 |
| Influenza A | 75+y | 0.009 | Jul 2022 |
| Influenza A | 75+y | 0.012 | Sep 2022 |
| Influenza A | 75+y | 0.022 | Oct 2022 |
| Influenza A | 75+y | 0.062 | Nov 2022 |
| Influenza A | 75+y | 0.268 | Dec 2022 |
| Influenza A | 75+y | 0.126 | Jan 2023 |
| Influenza A | 75+y | 0.005 | Feb 2023 |
| Influenza A | 75+y | 0.003 | Mar 2023 |
| Influenza A | 75+y | 0.017 | Apr 2023 |
| Influenza A | 75+y | 0.011 | May 2023 |
| Influenza A | 75+y | 0.006 | Jun 2023 |

RTI = Respiratory tract infections, hMPV = Human metapneumovirus; RSV = Respiratory syncytial virus

# Supplementary Table S7: Findings of sensitivity analysis conducted by limiting to episodes with specimen collected for viral testing between 3 days before and 3 days after the date of admission in older adults in Lothian by age bands and seasons

| **Age band** | **Season** | **Lab-confirmed hMPV hospital incidence (sensitivity analysis)** | **Lab-confirmed hMPV hospital incidence (main analysis)** | **Lab-confirmed RSV hospital incidence (sensitivity analysis)** | **Lab-confirmed RSV hospital incidence (main analysis)** | **Lab-confirmed Influenza A hospital incidence (sensitivity analysis)** | **Lab-confirmed Influenza A hospital incidence (main analysis)** |
| --- | --- | --- | --- | --- | --- | --- | --- |
| 60-74y | 2017/18 | 11.5 (6.2 - 18.4) | 11.5 (6.2 - 18.5) | 20.8 (13.1 - 28.4) | 20.8 (13.1 - 29.2) | 127.6 (109.2 - 148.3) | 129.1 (109.9 - 149.1) |
| 60-74y | 2018/19 | 33.9 (24.8 - 43.6) | 36.1 (26.3 - 46.7) | 34.6 (25.6 - 45.2) | 36.1 (26.3 - 46.7) | 102.3 (85.8 - 118.9) | 103.1 (85.8 - 119.7) |
| 60-74y | 2019/20 | 12.5 (7.3 - 18.4) | 12.5 (7.4 - 18.4) | 25.0 (17.7 - 33.9) | 25.8 (17.7 - 34.6) | 79.6 (65.6 - 94.3) | 79.6 (64.8 - 95.8) |
| 60-74y | 2020/21 | ^[[12]](#footnote-12)^ | * | * | * | * | * |
| 60-74y | 2021/22 | 6.5 (2.9 - 10.8) | 6.5 (2.9 - 11.5) | 7.2 (2.9 - 12.2) | 7.2 (3.6 - 12.2) | 14.4 (7.9 - 21.6) | 14.4 (8.6 - 20.2) |
| 60-74y | 2022/23 | 1.4 (0 - 3.5) | 1.4 (0 - 3.6) | 46.1 (35.5 - 57.5) | 46.2 (35.5 - 58.2) | 125.0 (107.2 - 142.7) | 130.7 (113.6 - 149.1) |
| ≥75y | 2017/18 | 41.7 (24.7 - 58.7) | 43.2 (29.3 - 60.2) | 72.6 (52.5 - 94.2) | 74.1 (52.5 - 95.8) | 511.2 (455.6 - 565.3) | 526.6 (474.1 - 579.1) |
| ≥75y | 2018/19 | 90.5 (69.4 - 114.6) | 92.0 (70.9 - 114.7) | 123.7 (96.5 - 152.4) | 123.7 (98.0 - 150.9) | 190.1 (158.4 - 226.2) | 191.6 (159.9 - 223.2) |
| ≥75y | 2019/20 | 25.4 (13.5 - 38.9) | 26.9 (15.0 - 38.9) | 92.7 (68.8 - 119.7) | 94.2 (71.8 - 118.1) | 269.1 (231.7 – 311.0) | 273.6 (236.2 - 312.5) |
| ≥75y | 2020/21 | * | * | * | * | * | * |
| ≥75y | 2021/22 | 4.2 (0 - 9.8) | 5.6 (1.4 - 11.2) | 19.5 (9.8 - 29.3) | 20.9 (11.2 - 32.1) | 40.4 (26.5 - 55.7) | 40.4 (26.5 - 55.7) |
| ≥75y | 2022/23 | 2.7 (0 - 6.8) | 2.7 (0 - 6.8) | 89.1 (67.5 - 110.7) | 91.8 (70.2 - 113.4) | 248.3 (215.9 - 282.1) | 252.4 (217.3 - 290.2) |

RTI = Respiratory tract infections, hMPV = Human metapneumovirus; RSV = Respiratory syncytial virus

# Supplementary Table S8: Characteristics of all-cause RTI, hMPV, RSV, and Influenza A hospital episodes in older adults in Scotland (2017-2023)

|  |  | **All-cause RTI**  **(n = 231,508)** | **hMPV**  **(n = 626)** | **RSV**  **(n = 1,841)** | **Influenza A (n = 7,408)** |
| --- | --- | --- | --- | --- | --- |
| **Age** | 60-74y | 86,896 (37.53%) | 269  (42.97%) | 758  (41.17%) | 3,296 (44.49%) |
|  | ≥75y | 144,612 (62.47%) | 357  (57.03%) | 1,083 (58.83%) | 4,112 (55.51%) |
| **Sex** | Female | 118,080 (51.00%) | 343  (54.79%) | 1,075 (58.39%) | 4,101 (55.36%) |
|  | Male | 113,428 (49.00%) | 283  (45.21%) | 766  (41.61%) | 3,307 (44.64%) |
| **Ethnicity** | White | 199,926 (86.36%) | 529  (84.50%) | 1,589 (86.31%) | 6,392 (86.29%) |
|  | Non-White | 29,593 (12.78%) | 95  (15.18%) | 240  (13.04%) | 942  (12.72%) |
|  | Data unavailable | 1,989  (0.86%) | 2  (0.32%) | 12  (0.65%) | 74  (1.00%) |
| **SIMD ^[[13]](#footnote-13)^** | 1 | 59,839 (25.85%) | 82  (13.10%) | 427  (23.19) | 1880 (25.38%) |
|  | 2 | 54,435 (23.51%) | 126  (20.3%) | 401  (21.78%) | 1716 (23.16%) |
|  | 3 | 46,114 (19.92%) | 144  (23.00%) | 376  (20.42%) | 1400 (18.90%) |
|  | 4 | 37,967 (16.40%) | 136  (21.73%) | 352  (19.12%) | 1257 (16.97%) |
|  | 5 | 32,378 (13.99%) | 134  (21.41%) | 274  (14.88%) | 1125 (15.19%) |
|  | Data unavailable | 775  (0.33%) | 4  (0.64%) | 11  (0.6%) | 30  (0.40%) |

RTI = respiratory tract infections, hMPV = human metapneumovirus, RSV = respiratory syncytial virus, SIMD = Scottish Index of Multiple Deprivation

The values in brackets indicate the percentage of the total cases reported in each column

# Supplementary Table S9: Severity and outcomes of hMPV-, RSV-, and Influenza A-associated RTI hospitalisations in older adults in Lothian and other health boards by age bands

|  |  | **Laboratory-confirmed hMPV in Lothian**  **(n = 204)** | **Laboratory-confirmed hMPV in other health boards**  **(n = 422)** | **Laboratory-confirmed hMPV in Scotland**  **(n = 626)** | **Laboratory-confirmed RSV in Lothian**  **(n = 461)** | **Laboratory-confirmed RSV in other health boards**  **(n = 1,380)** | **Laboratory-confirmed RSV in Scotland**  **(n = 1,841)** | **Laboratory-confirmed Influenza A in Lothian**  **(n = 1,484)** | **Laboratory-confirmed Influenza A in other health boards**  **(n = 5,924)** | **Laboratory-confirmed Influenza A in Scotland**  **(n = 7,408)** |
| --- | --- | --- | --- | --- | --- | --- | --- | --- | --- | --- |
| **Hospital LOS >5 days^[[14]](#footnote-14)^** | 60-74y | 9.89 (4.40 - 16.48) | 16.85 (11.80 - 22.47) | 14.50 (10.78 - 18.59) | 13.51 (8.65 - 18.38) | 9.95 (7.68 -12.57) | 10.82 (8.71 - 13.06) | 10.86 (8.59 - 13.45) | 8.55 (7.50 - 9.59) | 8.98 (8.07 -10.01) |
|  | ≥75y | 14.16 (7.96 - 21.24) | 14.34 (10.25 - 18.85) | 14.29 (10.92 - 18.21) | 13.41 (9.42 - 17.39) | 11.03 (9.05 - 13.26) | 11.63 (9.69 - 13.48) | 13.26 (11.07 - 15.57) | 12.85 (11.74 - 14.05) | 12.94 (11.96 - 13.96) |
|  | Total (≥60y) | 12.25 (8.32 - 17.26) | 15.40 (12.09 - 18.73) | 14.38 (11.66 - 17.25) | 13.45 (10.63 - 16.70) | 10.58 (8.91 - 12.17) | 11.30 (9.78 - 12.76) | 12.26 (10.58 - 13.95) | 10.90 (10.13 - 11.66) | 11.18 (10.46 - 11.93) |
| **ICU admissions** | 60-74y | 7.69 (2.20 - 13.21) | 19.10 (13.48 - 25.28) | 15.24 (11.15 - 19.71) | 10.81 (6.49 - 15.14) | 10.99 (8.38 - 13.61) | 10.95 (8.84 - 13.32) | 4.86 (3.24 - 6.65) | 8.73 (7.73 - 9.82) | 8.01 (7.13 - 9.01) |
|  | ≥75y | 3.54 (0.88 - 7.08) | 6.97 (4.09 - 9.85) | 5.88 (3.64 - 8.12) | 2.17 (0.72 - 3.99) | 5.20 (3.72 - 6.82) | 4.43 (3.32 - 5.54) | 1.73 (0.92 - 2.54) | 4.13 (3.45 - 4.84) | 3.62 (3.09 - 4.23) |
|  | Total (≥60y) | 5.39 (2.45 - 8.82) | 12.09 (9.24 - 15.40) | 9.90 (7.66 - 12.14) | 5.64 (3.69 - 7.59) | 7.61 (6.30 - 9.20) | 7.12 (6.03 - 8.26) | 3.03 (2.16 - 3.84) | 6.21 (5.64 - 6.85) | 5.58 (5.10 - 6.10) |
| **In-hospital deaths** | 60-74y | 1.10 (0 - 4.40) | 1.69 (0 - 3.93) | 1.49 (0.37 - 2.97) | 2.16 (0.54 - 4.32) | 2.97 (1.57 - 4.36) | 2.77 (1.72 - 3.96) | 2.11 (0.97 - 3.40) | 2.02 (1.49 - 2.58) | 2.03 (1.58 - 2.52) |
|  | ≥75y | 0.88 (0 - 2.65) | 6.15 (3.28 - 9.43) | 4.48 (2.52 - 6.72) | 2.17 (0.72 - 3.99) | 3.10 (1.98 - 4.34) | 2.86 (1.94 - 3.88) | 2.88 (1.73 - 4.04) | 2.87 (2.31 - 3.45) | 2.87 (2.36 - 3.40) |
|  | Total (≥60y) | 0.98 (0 - 2.45) | 4.27 (2.37 - 6.16) | 3.19 (1.92 - 4.63) | 2.17 (0.87 - 3.47) | 3.04 (2.17 - 3.91) | 2.82 (2.12 - 3.64) | 2.56 (1.75 - 3.37) | 2.48 (2.11 - 2.92) | 2.50 (2.16 - 2.85) |
| **Post-discharge deaths (up to 90 days***)* | 60-74y | 7.69 (2.20 - 13.19) | 8.99 (5.06 - 12.92) | 8.55 (5.58 - 12.27) | 8.11 (4.85 - 11.89) | 7.85 (5.76 - 9.95) | 7.92 (6.07 - 9.89) | 7.13 (5.19 - 9.08) | 6.20 (5.26 - 7.09) | 6.37 (5.52 - 7.16) |
|  | ≥75y | 10.62 (5.31 - 16.81) | 13.93 (9.84 - 18.44) | 12.89 (9.52 - 16.25) | 16.67 (12.68 - 21.39) | 13.38 (10.90 - 15.74) | 14.22 (12.19 - 16.53) | 11.19 (9.23 - 13.26) | 13.10 (12.02 - 14.33) | 12.69 (11.72 - 13.67) |
|  | Total (≥60y) | 9.31 (5.39 - 13.24) | 11.85 (9.00 - 14.93) | 11.02 (8.63 - 13.58) | 13.23 (10.41 - 16.05) | 11.09 (9.49 - 12.68) | 11.62 (10.21 - 13.2) | 9.50 (8.09 - 10.99) | 9.98 (9.22 - 10.70) | 9.88 (9.25 - 10.65) |

hMPV = Human metapneumovirus, RSV = Respiratory syncytial virus, LOS = length of stay, ICU = intensive care unit, CFR = case fatality rate

1. National Records of Scotland. Ethnic group, national identity, language and religion 2025 [Available from: <https://www.scotlandscensus.gov.uk/search-the-census#/topics/list?topic=Ethnic%20group,%20national%20identity,%20language%20and%20religion&categoryId=1> [↑](#footnote-ref-1)
2. Public Health Scotland. Deprivation: data 2025 [Available from: <https://www.scotpho.org.uk/wider-determinants/deprivation/data/>. [↑](#footnote-ref-2)
3. Lothian ranks 8th among 14 Scottish Health Boards for the proportion of its population in the 10% most deprived areas

   [↑](#footnote-ref-3)
4. The cylinders represent data sets.

   The blue boxes represent the variables in the dataset.

   Data linking is represented by red lines.

   The text in italics on or next to the red line presents the linking variables.

   The green boxes on or next to the red line represent the data cleaning procedure undertaken during linking.

   While linking laboratory data with RTI hospital admission data, the data were linked according to specimen collection date and hospital admission date first, before eliminating multiple tests associated with the same episode. [↑](#footnote-ref-4)
5. The Chi-square test was performed solely for all-cause RTIs to assess the statistical significance of demographic variable distributions in Lothian and other health boards. However, similar analyses were not conducted for virus-specific RTIs due to the lack of information on testing policies in other health boards [↑](#footnote-ref-5)
6. The Chi-square test showed a statistically significant distribution in the age distribution between all-cause RTIs in Lothian and other health boards (χ²=26.75, p<0.001) [↑](#footnote-ref-6)
7. The Chi-square test showed a statistically significant distribution in the ethnicity distribution between all-cause RTIs in Lothian and other health boards (χ²=2053.51, p<0.001) [↑](#footnote-ref-7)
8. The Chi-square test showed a statistically significant distribution in the SIMD distribution between all-cause RTIs in Lothian and other health boards (χ²=5317.39, p<0.001) [↑](#footnote-ref-8)
9. There were no laboratory-confirmed episodes in Lothian in the age band and season combination. As a result, extrapolation was not possible. [↑](#footnote-ref-9)
10. There were no laboratory-confirmed episodes in Lothian in the age band and season combination [↑](#footnote-ref-10)
11. There were no laboratory-confirmed episodes in Lothian in the age band and season combination. As a result, extrapolation was not possible [↑](#footnote-ref-11)
12. There were no lab-confirmed cases in Lothian in the age band and season combination [↑](#footnote-ref-12)
13. SIMD ranks data zones in Scotland in order of deprivation. The data are presented as SIMD quintiles with 1 indicating the most deprived area and higher quintiles progressively representing the less deprived areas [↑](#footnote-ref-13)
14. Estimates indicate the percentage of total cases requiring >5 days length of hospital stay along with 95% CI. [↑](#footnote-ref-14)
